# Natural Product-Derived Treatments for Attention-Deficit/Hyperactivity Disorder: Safety, Efficacy, and Therapeutic Potential of Combination Therapy

**DOI:** 10.1155/2016/1320423

**Published:** 2016-02-04

**Authors:** James Ahn, Hyung Seok Ahn, Jae Hoon Cheong, Ike dela Peña

**Affiliations:** ^1^Department of Pharmaceutical and Administrative Sciences, Loma Linda University, Loma Linda, CA 92350, USA; ^2^School of Medicine, Chungnam National University, Daejeon 301-747, Republic of Korea; ^3^Department of Pharmacy, Sahmyook University, Seoul 139-742, Republic of Korea

## Abstract

Typical treatment plans for attention-deficit/hyperactivity disorder (ADHD) utilize nonpharmacological (behavioral/psychosocial) and/or pharmacological interventions. Limited accessibility to behavioral therapies and concerns over adverse effects of pharmacological treatments prompted research for alternative ADHD therapies such as natural product-derived treatments and nutritional supplements. In this study, we reviewed the herbal preparations and nutritional supplements evaluated in clinical studies as potential ADHD treatments and discussed their performance with regard to safety and efficacy in clinical trials. We also discussed some evidence suggesting that adjunct treatment of these agents (with another botanical agent or pharmacological ADHD treatments) may be a promising approach to treat ADHD. The analysis indicated mixed findings with regard to efficacy of natural product-derived ADHD interventions. Nevertheless, these treatments were considered as a “safer” approach than conventional ADHD medications. More comprehensive and appropriately controlled clinical studies are required to fully ascertain efficacy and safety of natural product-derived ADHD treatments. Studies that replicate encouraging findings on the efficacy of combining botanical agents and nutritional supplements with other natural product-derived therapies and widely used ADHD medications are also warranted. In conclusion, the risk-benefit balance of natural product-derived ADHD treatments should be carefully monitored when used as standalone treatment or when combined with other conventional ADHD treatments.

## 1. Introduction

Attention-deficit/hyperactivity disorder (ADHD), a neurodevelopmental disorder characterized by the core symptoms of hyperactivity, inattentiveness, and impulsivity [1], is currently regarded as the most common neuropsychiatric disorder among children [2]. Furthermore, while this disorder is most often diagnosed during childhood, it may also affect an individual throughout life [3]. It is crucial to develop efficacious treatments for ADHD, given its serious academic, social, and familial consequences, along with the risk of incurring comorbid conditions and later substance abuse [4].

A number of treatment strategies have been suggested for ADHD since its recognition as a specific disorder in the 1970s. Presently, typical treatment plans utilize a nonpharmacological (behavioral/psychosocial) and pharmacological interventions or a combination of both [5]. A prominent nonpharmacological intervention in ADHD is behavioral therapy (behavior modification), which showed promise especially in youth and young adult ADHD patients. In principle, behavioral therapy operates via rewarding desired behaviors with positive reinforcement and discouraging problematic behaviors by introducing limits and consequences [6, 7]. Another type of behavior modification focuses on social skills training which is conducted in a group setting, where participants are taught by a therapist or qualified teacher appropriate/acceptable social behaviors which they (patients) are then encouraged to practice and repeat [7, 8]. Other approaches to ADHD therapy include memory training that employs computer software (Cogmed), neurofeedback, electroencephalography biofeedback, green space, meditation, yoga, exercise, and acupuncture in order to achieve symptom management (for reviews see [9–11]). However, as these therapies are not widely available, only a few patients can benefit from these treatment approaches. Although they are easy to implement, the need for time and professional therapists and also the involvement of not only the patient but also members of the family and teachers during the course of behavioral therapy limit the use of behavioral ADHD therapies.

ADHD has been associated with abnormalities in catecholaminergic function in the brain [12]. The use of medications that increase levels of catecholamine in the brain received widespread support as these drugs have been demonstrated to alleviate ADHD symptoms [12]. Drugs indicated in the management of ADHD are classified as stimulant and nonstimulant medications [13, 14]. The predominantly used or prescribed pharmacological interventions for ADHD are stimulant medications [13, 14]. Methylphenidate and dextroamphetamine are examples of these drugs, which are structurally similar to endogenous catecholamines and whose activity increases extracellular dopamine and norepinephrine levels, thereby correcting the underlying abnormalities in catecholaminergic functions and restoring neurotransmitter imbalance [12]. Nonstimulant alternatives include atomoxetine and norepinephrine specific reuptake inhibitors and the antidepressants bupropion, imipramine, and phenelzine [15, 16]. Nonstimulant alternatives have also been reported to increase catecholamine levels in the brain resulting in behavioral improvement. However, nonstimulant medications have been found to be inferior to stimulant treatments on efficacy endpoints [13, 16, 17].

While pharmacological treatments generally improve ADHD symptoms for most children, 20–30% of affected individuals are nonresponders or are unable to tolerate adverse side effects of these drugs [11, 18]. Some of the side effects of stimulant medications include headache, insomnia, and decreased appetite, motor tics, nausea, and abdominal pain [5, 15, 18]. Concerns over long-term exposure risks also discourage parents to medicate their children with stimulant drugs [17]. Furthermore, the high likelihood for dependence, diversion, and abuse, particular to drugs classified as schedule II (i.e., stimulants), limits the use of stimulant medications, as ADHD has been also associated with increased risk of substance use disorder [17].

Due to concerns over the safety and efficacy of current pharmacological ADHD interventions, there has been growing interest in the development of alternative treatments such as natural product-derived ADHD treatments including botanical or herbal medicines, vitamins, minerals, and amino acids [9–11]. These alternative treatments are appealing to parents who desire for more “natural” interventions for their children [9, 10]. Approximately 50% of parents of ADHD children used these treatments alone or in combination with other drugs or substances [19–22]. In this study, we provide a descriptive review of natural product-derived ADHD treatments including complementary ADHD interventions such as vitamins, minerals, and other nutritional supplements, report findings of clinical trials which evaluated efficacy and safety of these interventions and discuss the potential mechanism(s) by which these agents improve ADHD symptoms. The botanical agents are enumerated in Table 1, and Table 2 summarizes the vitamins, minerals, and other nutritional supplements evaluated for ADHD treatment. Furthermore, we also discuss the findings of studies which evaluated safety and efficacy of combining botanical agents or pharmacological ADHD treatments to treat ADHD. These information are summarized in Table 3.

## 2. Methods

Searches were made in the electronic databases PubMed, PyschINFO, and The Cochrane Library for English language articles from 2001 up to December 1, 2015. PubMed was used to search ADHD search terms in combination with particular natural product-derived treatments. The search strategy employed included combining these keywords: “natural products,” “plant”, “vitamins,” “minerals,” “essential fatty acids,” “amino acids”, “combination natural products,” or “combination with methylphenidate” and “ADHD” or “attention-deficit/hyperactivity disorder.” This process yielded 671 papers covering a wide range of research article types. As we were interested only in natural product-derived ADHD treatments which were evaluated in clinical trials, we concentrated on open-label, randomized controlled trials, as well as observational studies. Moreover, the inclusion criteria included samples consisting of children and adolescent ADHD patients (below 18 years of age), as well as sample size ≥ 10. The number of English language articles that were reviewed for this paper was 30.

## 3. Results

### 3.1. Pycnogenol® (French Maritime Pine Bark Extract)

Pycnogenol is a standardized extract derived from the bark of the French maritime pine (*Pinus pinaster*). This extract is rich in catechin, phenolic acids, procyanidins, and taxifolin, each with multiple biologic effects [30, 31]. Several studies on Pycnogenol showed its potentiality in improving ADHD symptoms in patients. Most notably, a double-blind, placebo-controlled trial with 61 participants (ages 6–14 years) reported that Pycnogenol treatment (1 mg/kg/day) for 1 month alleviated ADHD symptoms, particularly episodic hyperactivity and inattentiveness, and improved visual-motor coordination [30]. One month after termination of Pycnogenol, there was a relapse of symptoms in ADHD participants. The latter study also assumed that Pycnogenol's therapeutic benefits were mediated via an increase in nitric oxide production, which modulates dopamine and norepinephrine release and intake [30]. Pycnogenol reportedly produced mild side effects such as gastric discomfort. Nevertheless, the relatively small number of participants treated with Pycnogenol and the short duration of the study limit the generalization of the study's findings. It is noteworthy, however, that significant effects of Pycnogenol were observed, coupled with minimal side effects, supporting the suggestion that it could be used as an alternative ADHD treatment [30].

Another randomized, placebo-controlled study investigating efficacy of Pycnogenol reported improvement in attention along with reduction in oxidative DNA damage and normalization of homeostatic antioxidant status in ADHD patients treated for 1 month with the compound [31]. A month after Pycnogenol treatment, the total antioxidant status (TAS) was increased in ADHD children (ADHD children showed lower TAS levels at the beginning of the study compared with healthy controls) and was significantly elevated after 1 month of termination of Pycnogenol treatment. Oxidative stress is believed to be a contributing factor to the etiology of ADHD [52]. The improvement of ADHD symptoms in ADHD patients given Pycnogenol has been attributed to the drug's potent antioxidant effects [52]. Some ancillary benefits of Pycnogenol were normalization of the concentration of urinary catecholamines in children with ADHD and improvement in cerebral blood flow to regions of the brain implicated in this disorder [11, 53]. Of note, neither Pycnogenol nor the positive control, methylphenidate, outperformed placebo on any ADHD rating scale [54]. In summary, Pycnogenol is a promising botanical alternative in the management of ADHD symptoms, although more studies are required before it can be used as an ADHD treatment.

### 3.2. St. John's Wort

This herb “*Hypericum perforatum*,” while best known for its antidepressant qualities, was found to have beneficial effects on other psychiatric disorders, including obsessive compulsive disorder, major and bipolar depression, somatization disorder, and social phobia [55]. It has been suggested that the mechanism by which St. John's wort produces its therapeutic effects involves inhibition of the reuptake of dopamine, serotonin, and norepinephrine. A preliminary study reported improvement in ADHD symptoms of 3 ADHD patients (ages 14–16) given St. John's wort [56]. However, a more stringent randomized, double-blind, placebo-controlled trial found that 8 weeks of St. John's wort treatment (300 mg/day) did not alleviate ADHD symptoms in 54 ADHD patients (aged 6–17) [32]. In light of these findings, more studies are required to determine efficacy of St. John's wort in the treatment of ADHD. While the above studies did not report adverse effects of St. John's wort, investigations on the safety of this treatment are still necessary.

### 3.3. Ginseng

Ginseng contains ginsenosides, a class of phytochemicals with neuroprotective and antioxidant effects [26, 27]. Ginseng has also been reported to improve ADHD symptoms [27]. In addition, ginsenosides are reported to elevate levels of dopamine and norepinephrine; hence, they could potentially be used to treat ADHD. Ginseng's therapeutic benefits in ADHD were confirmed in an observational study involving ADHD participants (18 kids, ages 6–14) given Korean red ginseng (KRG,* Panax ginseng*) twice per day for 8 weeks (1,000 mg twice daily). In this study, KRG improved attention as measured by significant differences in omission errors measured by the computerized ADHD-diagnostic system (ADS) (78.56 ± 43.33 at baseline, 55.17 ± 21.44 at 8 weeks, *p* < 0.023) [26]. Omission errors in ADS measure inattentiveness. The significant decrease in omission errors has been associated with the restoration of impaired cognitive function in children with ADHD. Nevertheless, the small population size limits generalizability of the study's findings. A large-scale study with larger number of participants is necessary, as well as studies which assess long-term efficacy of KRG supplementation [27].

Another study (randomized, double-blind, and placebo-controlled) reported similar results in that participants (ADHD patients aged 6–15, *n* = 33), given one pouch of KRG (1 g KRG extract/pouch) twice per day, showed improvement in their inattention and hyperactivity scores after an 8-week treatment course compared with the control group (*n* = 37) [27]. Accordingly, the KRG group displayed significantly decreased inattention/hyperactivity scores compared with the control group at week 8 (least squared means of the differences in inattention adjusted for baseline scores are as follows: −2.25 versus −1.24, *p* = 0.048; hyperactivity: −1.53 versus −0.61, *p* = 0.047). They also showed decreased quantitative electroencephalography theta/beta ratio in comparison with the control group (least squared means of the differences are as follows: −0.94 versus −0.14, *p* = 0.001). The side effect profiles of ginseng included headache, fatigue, perspiration, and subjective issues with the taste of the ginseng product [26]. Ginseng's potential use as both alternative and adjunct ADHD treatment appears promising given the minimal safety concerns and remarkable efficacy.

### 3.4. *Ginkgo biloba*

A unique species of tree native to East Asia,* Ginkgo biloba, *has been extensively studied for its memory-enhancing effects [25]. Currently,* G. biloba* is used as an alternate treatment in patients with dementia or memory impairment [25, 49]. Studies also indicate that* G. biloba* may have therapeutic benefits in ADHD. For instance, Uebel-von Sandersleben et al. [24] reported improvement in overall quality of life, ADHD core symptoms, and Continuous Performance Test (CPT) performance in children given* G. biloba* (240 mg/daily) for 3–5 weeks. A reduced dose of* G. biloba* (50 mg), when combined with ginseng (200 mg) for 4 weeks of treatment, significantly improved ADHD symptoms in a test group of 36 children (ages 3–17) as quantified by Conners' Parent Rating Scale-Revised (long version) (CPRS-R [L]) [24]. Accordingly, there was significant improvement in each of the 3 areas which are most troublesome in ADHD (i.e., hyperactivity, cognitive problems, and oppositional behavior) in at least 50% of the subjects given* G. biloba* (50 mg) and ginseng (200 mg) up to 4 weeks after treatment. Reported side effects of* G. biloba* were observed; for example, subjects became more impulsive, hyperactive, aggressive, emotional, and tired and manifested increased sweating [24, 25].* G. biloba*'s beneficial effects have been linked to various activities such as improvement in cerebrovascular blood flow (alleviating hyperactivity), reversal of serotonergic (5-HT) 1A and noradrenergic receptor reductions, and inhibition of both monoamine oxidase- (MAO-) A and MAO-B in the brain [24, 25]. While* G. biloba* did produce improvement in ADHD symptoms, a trial by Salehi et al. [25] conducted over a 6-week period (double-blind, randomized, and placebo-controlled, *n* = 50 children) found that* G. biloba *(80–120 mg/day) was inferior to methylphenidate in efficacy endpoint. More formal clinical trials are required with longer duration and rigorous clinical endpoints in order to prove the worth of* G. biloba* in ADHD treatment.

### 3.5. Valerian

Valerian (*Valeriana officinalis*) is a perineal plant with sedative and antispasmodic effect. It has also been traditionally used in the treatment of insomnia, anxiety, and restlessness [9]. The efficacy of Valerian as an ADHD treatment has been evaluated in a double-blind, placebo-controlled pilot study [57]. Participants (30 kids, aged 5–11), given Valerian tincture three times a day for two weeks, showed improvement in ADHD symptoms, in particular, sustained inattention and impulsivity and/or hyperactivity [9, 33]. Nevertheless, the positive effects produced in the first two weeks of the study were not maintained overall following one week of no administration. The therapeutic effects of Valerian have been ascribed to valerenic acid (a significant component of Valerian) acting on the receptor gamma-aminobutyric acid (GABA)_A_ receptor [33]. GABA is the brain's main inhibitory neurotransmitter and has calming effects (for review see [28]). Deficiency in GABA causes anxiety, restlessness, and obsessive behavior, symptoms often seen in ADHD [58]. Valerian is considered generally safe and its use in children with ages 3–12 years was approved by the European Scientific Cooperative on Phytotherapy. However, Valerian must only be used under medical supervision [9, 10, 33]. More studies are required to augment limited clinical evidence supporting efficacy of Valerian in treating ADHD.

### 3.6. Ningdong

Ningdong granule (NDG) is a widely used Chinese medicinal preparation for various medicinal purposes. NDG showed promise in treating Tourette's syndrome, which invited studies to determine its efficacy in ADHD [28]. A randomized, double-blind, methylphenidate-controlled trial, where 72 children with ADHD were given 5 mg/kg/day of NDG (*n* = 36) or 1 mg/kg/day methylphenidate (*n* = 36) for a period of 8 weeks, reported that NDG was equally effective as methylphenidate in improving ADHD symptoms [28]. Accordingly, no significant difference was observed between the NDG and methylphenidate groups with regard to the data of teacher and parent ADHD rating scales at 8 weeks after medication. Furthermore, NDG produced less side effect profiles and was more tolerated by children as confirmed by urine, blood, and stool analysis along with renal and hepatic function tests. Interestingly, levels of homovanillic acid (HVA), which is involved in dopamine regulation, were increased in the sera of NDG-treated group with no resulting change in the concentration of dopamine. Hence the investigators proposed that NDG could be a safe, efficacious alternative treatment for ADHD [28]. One of the limitations of the study, however, involves the lack of placebo control in the study and the short-term outcomes. More meaningful pharmacological evidences to support the utility of NDG as an ADHD treatment are also required [28].

### 3.7. Bacopa

Bacopa (*Bacopa monnieri*) is an Ayurvedic medicine, also known as Brahmi or water hyssop. This natural remedy has been used for centuries to modulate memory, concentration, and learning [9]. Exploratory studies showed that Bacopa improves memory and learning in children with ADHD [9]. These findings were further supported by the findings of an open-label study which showed that Bacopa extract (225 mg/day, for 6 months) produced significant improvement in ADHD symptoms of participants (31 children, ages 6–12) [23]. In this study [29], the symptom scores for restlessness were reduced in 93% of children, while self-control was improved in 89% of ADHD participants. The attention-deficit symptoms were also attenuated in 85% of children. Moreover, learning problems, impulsivity, and psychiatric problems symptom scores were reduced for 78%, 67%, and 52% of children, respectively. It was further reported that 74% of the children exhibited up to a 20% reduction, while 26% of children displayed between 21% and 50% reduction in the total subtests scores. The efficacy of Bacopa in this context has been attributed to its neuroprotective and antioxidant effects, as well as regulation of dopamine, and inhibition of cholinesterase [9, 23]. Some minor gastrointestinal side effects were reported with the use of Bacopa, although it was well tolerated by children [23]. Further studies are warranted to confirm the safety and efficacy of this botanical agent when used as an ADHD treatment.

### 3.8. Passion Flower

Passion flower is comprised of the fragmented or cut, dried aerial parts of* Passiflora incarnata *L., which is a traditional remedy for anxiety and ADHD [29, 59]. Effect of passion flower in alleviating ADHD symptoms was tested in 34 children with ADHD randomized to receive tablets of* Passiflora* (0.04 mg/kg/day, twice daily) or methylphenidate (1 mg/kg/day, twice daily), dosed on a weight-adjusted basis, for 8 weeks. Both parent and teacher rating scores revealed no significant difference in the clinical benefits of* Passiflora* and methylphenidate treatment in ADHD children over the course of the trial (*F* = 0.007, df = 1, and *p* = 0.93; and *F* = 0.006, df = 1, and *p* = 0.94, resp.). Moreover, side effect profile of* Passiflora* was less compared with methylphenidate [59]. As the study was conducted in a small population of patients, the results of this study need to be validated in larger trials.

### 3.9. Emerging Natural Product-Derived ADHD Treatments: Evidence from Preclinical Studies

#### 3.9.1. Oroxylin A

Oroxylin A (5,7-dihydroxy-6-methoxyflavone) is a flavonoid isolated from the root of* Scutellaria baicalensis *Georgi, a herb commonly found in East Asia [60]. Oroxylin A is an antagonist of the GABA_A_ receptor [60]. Furthermore, its biological activities, including antioxidant, anti-inflammatory, and antiallergy as well as memory-enhancing and neuroprotective effects, provide basis for its potential therapeutic use in ADHD. Preclinical studies showed that Oroxylin A or its derivative (5,7-dihydroxy-6-methoxy-4′-phenoxyflavone) produced improvement of ADHD-like behaviors in spontaneously hypertensive rats, animal models of ADHD [61, 62]. The therapeutic activities of Oroxylin A have been ascribed to enhanced dopamine neurotransmission. Ongoing studies are investigating efficacy of Oroxylin A in ADHD patients.

#### 3.9.2. YY162

YY162 is a combination pharmaceutical product consisting of terpenoid-strengthened* G. biloba* and ginsenoside Rg3 from ginseng. A recent study showed improvement of ADHD-like symptoms, induced by Aroclor1254, in mice given YY1612 [63]. The degree of alleviation of ADHD-like symptoms induced by YY1612 was found to be comparable to that exerted by methylphenidate. YY1612 also produced neuroprotective effects with minimal behavioral side effects. The mediation of the ADHD-like symptoms in mice by YY1612 is believed to be due to its antioxidant properties and its ability to regulate and control dopamine and norepinephrine transporters [63]. Further studies are required to show the potentiality of YY1612 as an ADHD medication.

#### 3.9.3. *Sideritis scardica*

The genus* Sideritis* plant, species “*Sideritis scardica,*” has been used traditionally in the Mediterranean region as teas and flavoring agents and also for treatment purposes [57]. Studies have shown that* S. scardica* extracts may have an inhibitory effect on the reuptake of three key monoamines: dopamine, serotonin, and noradrenaline [57]. Moreover, an electroencephalogram (EEG) study showed that* Sideritis* treatment in rats induced frequency patterns comparable to those produced by methylphenidate [64]. Overall, these studies suggest the benefit of* Sideritis* in the treatment of mental disorders, including ADHD. Further in vivo studies measuring its efficacy in ADHD are warranted.

#### 3.9.4. Rhodiola

Rhodiola (*Rhodiola rosea*) has been shown to stimulate CNS activity and exert adaptogenic and neuroprotective effects [65]. The antifatigue and antianxiety effects of Rhodiola extract have been demonstrated in various clinical trials [66, 67]. In view of these results, Rhodiola may have therapeutic potential for treating anxiety disorders and depression [68, 69]. No adverse side effects were reported in the above-mentioned clinical trials making it a potentially safe medication. Preclinical studies reported that Rhodiola may enhance levels of serotonin by increasing the transport of serotonin precursors (e.g., tryptophan and 5-HTP) [70]. Rhodiola also appears to inhibit the activity of acetylcholinesterase, an enzyme which degrades acetylcholine [71]. These properties of Rhodiola demonstrate its potential as an ADHD treatment. No trials have been conducted to test efficacy of Rhodiola in ADHD.

### 3.10. Nutritional Medicines and Supplements

Previous studies showed that certain vitamins, minerals, and amino acids may contribute to the pathology of ADHD (discussed below). Thus, a wide range of nutritional supplements (vitamins and minerals) have been proposed as potential adjunct and alternative ADHD treatments. Because these agents are closer to food substances than drugs, they do not have similar rigorous restrictions by the Federal Drug Administration that drugs do have and can be purchased over the counter [45].

#### 3.10.1. Vitamins

Vitamins have been used as potential adjuncts or alternative treatments for ADHD based on anecdotal evidence that they produced improvement in attentiveness and concentration in normal children [10]. As an example, combining magnesium (6 mg/kg/day) with vitamin B6 (0.6 mg/kg/day) during an 8-week treatment course improved ADHD symptoms in children [45], with symptoms recurring again once supplementation was discontinued. The beneficial attributes of vitamin B6 on ADHD are attributed to its ability to influence the production of serotonin [9, 58].

The effects of vitamin C treatment with alpha linolenic acid- (ALA-) rich nutritional supplementation in the form of flax oil on blood fatty acids composition and behavior in children with ADHD were also examined [72]. This study found that red blood cell membrane fatty acids were significantly improved in ADHD patients receiving the above supplementation and alleviated ADHD symptoms in ADHD patients as evidenced by reduction of hyperactivity scores [72]. As mentioned above, oxidative stress has been postulated to play a role in ADHD [52, 73]. Thus, the antioxidant effect of vitamin C may have contributed to the beneficial effect of this supplementation regimen in ADHD children, along with effects of ALA (ALA is a precursor fatty acid and with elongation and unsaturation gets converted to docosahexaenoic acid which is critical for normal brain development) [73, 74].

Currently, there is another ongoing clinical study evaluating the effect of tocotrienols for children with ADHD, of which findings are not yet reported: Tocotrienols for School-going Children With ADHD (TOCAT). Tocotrienol is a form of vitamin E which is described to exert antioxidative properties [74]. Moreover, aside from antioxidant properties, tocotrienol may also inhibit phospholipase A2 enzyme which is involved in metabolism of polyunsaturated fatty acids, which is purportedly dysfunctional in ADHD [74, 75].

While vitamins may not directly affect symptoms of ADHD, they have the added benefit of replenishing any deficiencies due to poor dietary habits [10]. Nevertheless, when initiating megadoses (i.e., 100 times the recommended daily intake) of vitamins [76], caution must be used in younger patients as evidence supporting efficacy of vitamins remains to be established. Moreover, megadoses of vitamins were sometimes detrimental at such high doses [10, 76, 77]. Further studies (randomized, double-blind, and placebo-controlled) are necessary to validate the use of vitamins to treat ADHD.

#### 3.10.2. Minerals

Another proposed alternative intervention for ADHD is mineral supplementation. Mineral deficiencies have also been implicated in the etiology of this disorder, making supplementation a potential means of improving ADHD symptoms. Minerals, as cofactors, have a role in the synthesis, uptake, and breakdown of crucial neurotransmitters associated with ADHD [11, 76, 78]. Moreover, minerals such as magnesium and calcium are necessary for aerobic metabolism and serve as cofactors in the degradation of blood glucose through glycogenesis, the citric acid cycle and respiratory chain in the mitochondria. Enhanced energy metabolism of neurons and glial cells, regulated by the mitochondria, is largely dependent on the presence of minerals as well as vitamins (for review see [76]).

A 12-week double-blind study found that children supplemented with zinc sulfate (150 mg) showed reduced impulsiveness, hyperactivity, and socialization difficulties [46]. A study by Akhondzadeh et al. [47] also reported alleviation of ADHD symptoms in children given zinc sulfate along with methylphenidate therapy. The side effect profile proved minimal, with gastrointestinal discomfort and metallic taste being the most common. When zinc levels are low, corresponding impairments in cognitive functions may ensue [11, 47]. Thus, it is believed that zinc supplementation would have beneficial effects on cognitive functions. However, another study showed that zinc supplementation produced negligible beneficial effects on relieving ADHD symptoms [48]. These opposing clinical outcomes can be attributed to genetic factors, dosage differences, and nutritional status of patients [11].

Iron is another well-studied mineral that has undergone clinical trial for the treatment of ADHD. Iron is a cofactor in the synthesis of both norepinephrine and dopamine [11, 18]. As shown in previous studies, anemic children (iron deficient) exhibited attentional deficits [78]. A randomized, double-blind, placebo-controlled trial found that iron supplementation in children with ADHD (23 kids, ages 5–8) proved beneficial in relieving ADHD symptoms [44]. Notably, supplementation in children without iron-deficiency anemia had inconsistent, variable results [11, 78].

Another mineral shown to improve ADHD symptoms is magnesium. The involvement of this mineral in neurotransmitter synthesis supports its potentiality as an ADHD treatment [79]. In a previous study, children supplemented with magnesium and vitamin B6 showed improvement in their ADHD symptoms [45].

In general, these findings indicate the worth of mineral supplementation in ADHD. Nevertheless, a strategy suggested to advance the use of minerals (as well as vitamins) in ADHD treatment is to combine these nutrients to adequately affect the complicated biochemical pathways that may be defective in ADHD patients [76] and to mimic the vast array of nutrients required for optimal brain functioning [76]. In an open-label, on-off-on-off (reversal design) study involving 14 ADHD children (8–12 years old) treated with a 36-ingredient micronutrient (vitamins and minerals) titrated up to maximum dose (15 capsules/day) for 8 weeks, withdrawn for 4 weeks and reinstated for a further 8 weeks and withdrawn again for 4 weeks, improvement in ADHD symptoms and mood, as well as enhanced overall functioning during treatment phases, with deterioration in ADHD symptoms, mood, and overall functioning during the withdrawal phases, was observed in ADHD participants [80]. Further statistical analyses also confirmed clinically and statistically significant change between the intervention and withdrawal phases, with large effect sizes observed before to after exposure of micronutrients (*d* = 1.2–2.2) on ADHD symptoms during intervention phases [80]. This study also found that 71% of participants showed at least a 30% decrease in ADHD symptoms by the end of the second treatment phase, and 79% were identified as “much improved” or “very much improved” at the end of the second phase (5 months) based on the clinician-rated Clinical Global Impressions (CGI) Scale when evaluating functioning generally. The Strengths and Difficulties Questionnaire (SDQ)—parent version—also revealed that these beneficial effects of micronutrients occurred across other areas of functioning including emotional symptoms, conduct problems, and prosocial behaviors. The children's self-reports also verified the improvements. There was also remarkable adherence to treatment, and side effects were mild and transitory without safety issues after blood analysis of participants. Indeed, a combination of different micronutrients may be more feasible and produce more significant clinical benefits compared with treatment with a single micronutrient only [76, 80].

#### 3.10.3. Amino Acids

A number of amino acids have been shown to exert direct or indirect effects on the levels of specific neurotransmitters. Thus, they have the potential to be used in treating ADHD. Amino acids, glycine, L-theanine, L-tyrosine, taurine, acetyl-L-carnitine (ALC), GABA, 5-hydroxytryptophan (5-HTP), and s-adenosyl-L-methionine (SAMe), are all considered potential complementary ADHD interventions [9, 10]. A significant portion of studies on amino acid supplementation has focused on ALC, an amino acid derivative. One such study (randomized, double-blind, and placebo-controlled), utilizing ALC, reported that supplementation with this protein derivative significantly reduced symptoms of ADHD, in particular, hyperactivity and poor social behavior, in trial participants (51 children, ages 6–13) [34]. This effect of ALC has been attributed to modulation of neural transmission by increasing acetylcholine synthesis, stimulating its release and release of dopamine in the striatum in various brain regions, other than carnitine metabolism [34]. On the other hand, a randomized, double-blind placebo-controlled study reported conflicting findings in that no significant effects of ALC were observed in ADHD patients (112 children, ages 5–12) [35].

Theanine is an amino acid found in both green and black teas [81]. This nonproteinaceous component (n-ethylglutamic acid) has garnered increasing attention recently due to its purported central nervous system effects. Because of its ability to cross the blood-brain barrier, theanine has a variety of pharmacological effects, most pertinent of which is anxiolytic effect. These effects of theanine have been attributed to regulation of dopamine and serotonin and an increased production of inhibitory neurotransmitters [81]. Additionally, it has been reported that theanine produced improvement in selective attention during the execution of mental tasks via modulation of alpha brain wave activity. Currently there are a handful of studies examining the therapeutic potentials of theanine in ADHD (for review see [81]). Theanine has also been suggested for panic disorder, bipolar disorder, and obsessive compulsive disorder, aside from ADHD and anxiety disorders.

#### 3.10.4. Essential Fatty Acids

The effects of essential fatty acids (EFAs, e.g., omega-3 and omega-6) in treating ADHD in children have been recently investigated. Supplementation with these fatty acids has shown modest success in controlling ADHD symptoms [82, 83]. A study by Richardson and Puri [36] reported that ADHD children showed improved attention and reduced hyperactive and defiant behaviors after supplementation with highly unsaturated fatty acids (comprised of eicosapentaenoic acid (EPA) 186 mg/day, docosahexaenoic acid (DHA) 480 mg/day, *γ*-linolenic acid 96 mg, vitamin E 60 IU, cis-linoleic acid 864 mg, AA 42 mg, and thyme oil 8 mg). The influence of these fatty acids on signal transduction relevant to neuronal structure, development, and functions may play a role in EFA-induced improvement of ADHD symptoms [36]. Another study revealed improvement in inattention and oppositional behaviors in children who received combined EFA supplementation (polyunsaturated fatty acid (PUFA) supplement comprised of 480 mg DHA, 80 mg EPA, 40 mg arachidonic acid (AA), 96 mg gamma-linolenic acid (GLA), and 24 mg alpha-tocopheryl acetate), although not all ADHD behaviors were alleviated by this treatment regimen [37]. Furthermore, Sinn and Bryan [38] reported marked improvement in ADHD symptoms in children supplemented for 15 weeks with EFA as opposed to those receiving placebo. The same supplementation regimen also improved attention control and vocabulary performance in ADHD children. Manor et al. [40] also reported improvement of ADHD symptoms (impulsivity, inattention) and mood and behavioral problems in ADHD patients given phosphatidylserine containing omega-3, EPA, and DHA [40]. The exact mechanism by which EFAs benefit ADHD is still unresolved but may be associated with the role of EFAs in brain development (e.g., effects on gene expression, neural signaling, and cellular growth and functions) [10, 37–40, 82]. Another proposed mechanism suggests that these therapeutic benefits may arise from increased dopaminergic and serotonergic activity as a result of elevated EFAs [11, 40, 82].

In contrast, other studies including randomized clinical trials reported no significant effects/benefits in ADHD patients treated with EFAs compared with the placebo group [41–43]. A meta-analysis of randomized, controlled trials with EFAs revealed disappointing results in that most of these trials do not support robust clinical effect of EFA supplements as a treatment for children with ADHD [84]. Recent reviews also reported modest benefits of EFA supplementation in ADHD patients [85–87]. In summary, although some studies have reported therapeutic benefits of EFA supplementation, the current evidence for EFA as a complementary and alternative medicine for ADHD remains controversial [86].

## 4. Combination Treatment Approaches: Effects of Two Botanical Agents or When Given with an ADHD Drug

Given the multifactorial characteristic of ADHD, the management of this disorder may benefit from a multimodal approach. Presently, ADHD management trends are favoring treatment of ADHD with a combination of various treatment approaches [88, 89]. Multimodal strategies are highly attractive because they are more “holistic” and patient specific. Moreover, combined therapies may also help improve overall functioning by targeting symptoms of comorbid disorders such as substance use disorder, compulsive disorders, and learning disabilities [58].

Most multimodal treatment approaches employ the use of stimulant medications, given their wide use in managing ADHD, and behavioral/psychosocial therapy. A multisite clinical trial, the Multimodal Treatment of ADHD (MTA) study, revealed that combination (medicinal and behavioral treatment) and medicinal management (methylphenidate) interventions were significantly superior to behavioral or community care alone for managing ADHD symptoms [88]. Moreover, there was a perceived advantage of combination treatment over single treatment (medicinal and behavioral) for managing other functioning domains such as social skills, academics, oppositional behavior, and anxiety/depression [89].

In contrast, only a few studies have evaluated combination therapy utilizing nutritional/botanical supplements with behavioral therapy or pharmacological ADHD agents (Table 3). What is more, there are limited studies which screened therapeutic potential of combining a botanical agent with another plant-derived product or nutritional supplements. Nevertheless, the findings of these studies have been encouraging. Discussed below are some of these landmark studies.

### 4.1. Efficacy of Combinatorial Natural Product-Derived Treatment and Pharmacological ADHD Therapy

Two Chinese medicine herbal treatments have been previously evaluated as adjunct treatments to methylphenidate. A 2-week trial in children randomized to receive Yizhi mixture (a combination of 10 herbs designed to affect Yin/Yang liver functions), methylphenidate, or combination treatment reported more significant improvement of ADHD symptoms in children subjected to combination treatment than in those randomized to either individual treatment. There were also fewer side effects in children given Yizhi mixture alone or combination treatment than in those assigned to the methylphenidate group [51]. In another study, efficacy of combination therapy with Jingling oral liquid and methylphenidate was tested [50]. This study showed that children randomized to this treatment approach showed greater improvement in ADHD symptoms and well as tic symptoms compared to treatment with methylphenidate only. What deserves further investigation is the safety profile of these herbal treatments, given alone or in combination with methylphenidate.

In another study, Akhondzadeh et al. [47] performed a randomized, double-blind trial to examine the potential benefits of a zinc sulfate treatment alongside methylphenidate. The result showed that methylphenidate therapy was enhanced with the addition of zinc supplementation in participants (children, ages 5–11). Therefore, combining botanical agents or nutritional supplements with pharmacological ADHD treatment may be a promising ADHD treatment approach. As expected with combinatorial treatment approaches, combination therapy may enhance therapeutic efficacy or overcome therapeutic limitations of individual treatments.

### 4.2. Efficacy of Combining Two Botanical Agents

The efficacy of combining American ginseng extract,* Panax quinquefolium* (200 mg), and* Ginkgo biloba* extracts (50 mg) to alleviate ADHD symptoms was evaluated in 36 children aged 3–17 years [24]. Results of this study indicated that, after 4 weeks of treatment with this mixture, 50% of the subjects showed improvement in each of the 3 areas that are most troublesome in ADHD, namely, hyperactivity, cognitive problems, and oppositional behavior. The diverse mechanisms of these agents including their ability to enhance brain functions may have been responsible for the efficacy of this combined therapy [49]. Nevertheless, well-controlled trials with rigorous clinical endpoints need to be undertaken before drawing definitive conclusions on the safety and efficacy of this treatment approach.

## 5. Conclusion

There are a number of available treatment options for ADHD; however, some of them may pose risks to patients [10]. The botanical agents discussed in this study appear to be promising ADHD treatments considering their therapeutic effects and negligible negative side effects. Nevertheless, it has to be noted that ADHD is a complex disorder having multiple causes and, thus, the use of natural product-derived treatments alone may not sufficiently affect consistent change in ADHD symptoms (see [76]). As mentioned previously, more pronounced clinical benefit may be achieved by employing a multimodal treatment approach such as combination therapy of different botanical agents and/or micronutrients, botanical agents and conventional pharmacological treatments, and also behavioral therapy.

Although the use of natural medications for ADHD has been considered as a “safer” approach, natural products are still far from being called as standard ADHD treatments due to the lack of comprehensive and appropriately controlled clinical studies that interrogate both their efficacy and safety. Moreover, it is challenging to compare efficacy profiles of herb therapy with conventional pharmacological ADHD treatments, mainly because herbal preparations are not standardized, and question regarding their purity, reliability, safety, and toxicity profiles will always arise [58]. Therefore, using pure medications with known doses, described mechanisms of action, and adverse effects profiles is preferable with regard to the use of natural product-derived ADHD treatments.

The findings from recent, albeit few, studies which evaluated efficacy of adjunct therapy of botanical agents and nutritional supplements with a pharmacological ADHD treatment or another botanical agent suggest that combination therapy may be a promising approach in ADHD treatment. Nevertheless, positive findings from above-mentioned studies need to be replicated, and evidence for long-term effectiveness and safety should be aptly demonstrated. Efficacy of combining other botanical agents with pharmacological agents including other medications aside from methylphenidate (e.g., atomoxetine, guanfacine, and clonidine) or with behavioral therapy should also be explored in future studies. As herbs usually contain more than one psychoactive substance and may have additive or interactive effects with the combined treatment, the risk-benefit balance of natural product-derived ADHD treatments should be carefully considered when combined with other medications.

## Figures and Tables

**Table 1:** Clinical trials evaluating safety and efficacy of botanical agents for ADHD.

Study	Botanical agent	Method	Participants	Outcomes	Proposed mechanism of action	Comments (side effects, etc.)
Dave et al. [23]	*Bacopa* (*Bacopa monnieri*) Standardized *Bacopa monnieri* extract (SBME) (225 mg/day), for 6 months	Open-label study	31 children, 6–12 years old with ADHD	Reduction of ADHD symptoms (restlessness, poor self-control, inattention, impulsivity, etc.)	Neuroprotection, regulation of dopamine, and inhibition of cholinesterase	Safe and well tolerated Mild gastrointestinal side effects

Uebel-von Sandersleben et al. [24]	*Ginkgo biloba* Ginkgo (EGb 761®), 240 mg daily, given for 3 to 5 weeks	Open clinical pilot study	20 children with ADHD	Improvement of ADHD core symptoms	Improvement in cerebrovascular blood flow, reversal of 5-HT1, and noradrenergic receptor reductions Reverse inhibition of MAO-A and MAO-B	Very low rates of mild adverse events during observational period
Salehi et al. [25]	*Ginkgo biloba* *Ginkgo biloba* (80–120 mg/day) or methylphenidate (20–30 mg/day), for 6 weeks	Randomized, double-blind controlled trial	50 children, 6–14 years old with ADHD (*n* = 25* Ginkgo biloba* versus *n* = 25 methylphenidate)	Improvement of ADHD symptoms. Less effective than methylphenidate		Lesser side effects (headache, insomnia, and loss of appetite) than methylphenidate

Lee et al. [26]	*Ginseng* Korean red ginseng (1,000 mg b.i.d.) and placebo twice a day for 8 weeks	Observational study	18 children, 6–14 years old with ADHD	Improvement in attention	Nootropic effect on CNS Increased dopamine and norepinephrine levels Neuroprotective effects	Taste aversion and repulsion to ginseng
Ko et al. [27]	*Ginseng* Korean red ginseng extract (1 g KRG extract/pouch) twice a day for 8 weeks	Randomized, double-blind, placebo- controlled trial	70 children, 6–15 years old with ADHD (*n* = 33 KRG versus *n* = 37 placebo)	Improvement of hyperactivity and inattention symptoms Decreased quantitative electroencephalography theta/beta ratio		No reported adverse events/side effects

Li et al. [28]	*Ningdong* Ningdong (5 mg/kg/day) versus methylphenidate (1 mg/kg/day), for 8 weeks	Randomized, double-blind, methylphenidate-controlled trial	72 children, 6–13 years old with ADHD (*n* = 36 Ningdong versus *n* = 36 methylphenidate)	Similar efficacy to control (methylphenidate)	Regulation of dopamine by increasing HVA concentration in the sera	Hypersomnia

Akhondzadeh et al. [29]	*Passion flower* *Passiflora incarnata*	Double-blind, randomized, methylphenidate-controlled clinical trial	34 children with ADHD	Improvement of ADHD symptoms	Not specified	Decreased appetite and anxiety/nervousness compared with methylphenidate group

Trebatická et al. [30]	*Pycnogenol* Pycnogenol (1 mg/kg/day) or placebo treatment for 4 weeks	Randomized, double-blind, placebo-controlled study	61 children, 6–14 years old with ADHD (*n* = 44 Pycnogenol, versus *n* = 17 placebo)	Attenuation of hyperactivity and improvement of attention, visual-motoric coordination, and concentration	Influence on catecholamine formation or metabolism Increased production of nitric oxide which modulates dopamine and norepinephrine release and intake	Mild side effects including slowness and gastric discomfort

Chovanová et al. [31]	*Pycnogenol* Pycnogenol (1 mg/kg/day) or placebo treatment for 4 weeks	Randomized, double-blind, placebo-controlled study	61 outpatient children, 6–14 years old with ADHD (*n* = unspecified Pycnogenol versus placebo)	Improved attention, reduction in oxidative damage	Antioxidant properties	No reported adverse events/side effects

Weber et al. [32]	*St. John's wort*	Randomized, double-blind, placebo-controlled study	56 children, 6–17 years old with ADHD (*n* = 27 SJW versus *n* = 27 placebo)	No significant improvement in ADHD symptoms		No reported adverse events/side effects

Razlog et al. [33]	*Valerian (Valeriana officinalis)*	Double-blind, placebo- controlled, clinical trial	30 children, 5–11 years old with ADHD (*n* = 10*Valeriana officinalis* mother tincture (VOMT) or *n* = 10 3x potency of VOMT versus *n* = 10 placebo, for 3 weeks)	Improvement of ADHD symptoms in VOMT or 3x potency group, in comparison to placebo, in particular, inattention, impulsivity, and/or hyperactivity	Inhibition of the breakdown of GABA in the central nervous system	No reported adverse events/side effects

**Table 2:** Clinical trials evaluating safety and efficacy of nutritional supplements for ADHD.

Study	Supplement	Method	Participants	Outcomes	Proposed mechanism of action	Comments (side effects, etc)
Torrioli et al. [34]	*Acetyl-L-carnitine (LAC)* LAC (500 mg, 2 times/day) or placebo for 12 months	Randomized, double-blind, placebo- controlled, parallel, multicenter study	51 children (ADHD and Fragile X syndrome), 6–13 years old (*n* = 24 ALC versus *n* = 27 placebo)	Reduction of ADHD symptoms versus Placebo on Clinical Global Impressions Parental Rating	Modulation of neural transmission by increasing acetylcholine synthesis, stimulating its release and release of dopamine in the striatum in various brain regions	No adverse events/side effects reported

Arnold et al. [35]	*Acetyl-L-carnitine (ALC)* ALC in weight-based doses from 500 to 1,500 mg b.i.d. or placebo for 16 weeks	Multisite parallel-group double-blind randomized pilot trial	112 children, 5–12 years old (*n* = 53 Acetyl-L-carnitine versus *n* = 59 placebo)	Acetyl-L-carnitine superior to placebo in inattentive subtype		No adverse events/side effects reported

Richardson and Puri [36]	*Essential fatty acids* Highly unsaturated fatty acid (HUFA): EPA 186 mg/day, DHA 480 mg/day, *γ*-linolenic acid 96 mg, vitamin E 60 IU, cis-linoleic acid 864 mg, AA 42 mg, and thyme oil 8 mg or olive oil (placebo), for 12 weeks	Randomized, double-blind, placebo- controlled study	41 children, 9 participants withdrew before the end of 12-week period, 8–12 years old (*n* = 15 HUFA versus *n* = 14 placebo)	Attenuation of ADHD symptoms, for example, inattention, hyperactivity, improvement in cognition and emotion	Influence on signal transduction relevant to neuronal structure, development, and functions	Upset stomach and difficulty swallowing

Stevens et al. [37]	*Essential fatty acids* PUFA supplement comprised of 480 mg DHA, 80 mg EPA, 40 mg arachidonic acid (AA), 96 mg GLA, and 24 mg alpha-tocopheryl acetate or an olive oil placebo for 4 months	Randomized, double-blind, placebo- controlled study	50 children (girls and boys), *n* = 25 PUFA supplementation, *n* = 25 placebo	Clear benefit for all behaviors characteristic of ADHD was not observed Treatment effects for conduct and attention as well as with clinical improvements in oppositional/defiant behavior	Mediation of abnormal neuronal signaling that results in aberrant behaviors	Not specified

Sinn and Bryan [38]	*Essential fatty acids* LC-PUFA capsules containing 400 mg fish oil and 100 mg evening primrose oil with EPA (93 mg), DHA (29 mg), GLA (10 mg), and vitamin E (1.8 mg) or placebo. Six active or 6 placebo capsules per day for 15 weeks	Randomized, double-blind, crossover, placebo-controlled study	132 children (data available for 104 and 87 children) 7–12 years old (*n* = 36 PUFAs versus *n* = 41 PUFA + micronutrients versus *n* = 27 placebo)	Significant treatment effects based on parental rating of core ADHD symptoms in both PUFA groups versus placebo	Modulation of neural cell signaling and neurotransmitter processes PUFAs with other nutrients such as vit. C, B_3_, and B_6_ modulates PUFA's role in the synthesis of prostaglandins and chemicals important for biological and brain function	No adverse events/side effects

Sinn et al. [39]	*Essential fatty acids* LC-PUFA capsules containing 400 mg fish oil and 100 mg evening primrose oil with EPA (93 mg), DHA (29 mg), GLA (10 mg), and vitamin E (1.8 mg) or placebo. Six active or 6 placebo capsules per day for 15 weeks	Randomized, one-way crossover, placebo- controlled study Phases 1 and 2	Phase 1: *n* = 129 children with ADHD (PUFA versus PUFA + multivitamins/minerals versus placebo for 15 weeks) Phase 2: *n* = 104 children with ADHD (PUFA, PUFA + multivitamin/minerals, and placebo for 15 weeks)	Improved ability on attention control and vocabulary performance during phase 2	Influence on metabolic and neural activities Increasing dopamine activity in the frontal lobe	Two cases of nausea and one episode of nose bleed

Manor et al. [40]	*Essential fatty acids* 2 capsules twice a day of phosphatidylserine (PS) containing omega-3 (300 mg of PS and 120 mg of EPA + DHA) or cellulose capsules as placebo, for 15 weeks	Randomized, double-blind, single-center, placebo- controlled trial	200 children (6–13 years old) randomly assigned to PS-omega-3 capsules or placebo *N* = 162 children completed 15 weeks of treatment (*n* = 110PS-omega-3, *n* = 52 placebo)	Improvement of ADHD symptoms (impulsivity, inattention, mood, and behavior issue)	Maintenance of integrity of cell membranes Influence on dopaminergic and cholinergic systems Increasing omega-3 LC-PUFA which improves behavioral, sensory, and neurological dysfunction	Mild adverse event profile: GI discomfort, atopic dermatitis, nausea, tics, and hyperactivity

Raz et al. [41]	*Essential fatty acids* EFA capsules containing 240 mg of linoleic acid (LA) 60 mg of alpha-linolenic acid (ALA), 95 mg of mineral oil, and 5 mg of a-tocopherol (as an antioxidant) 2 times/day or placebo: vit. C (500 mg ascorbic acid) 2 times/day, for 7 weeks	Randomized, double-blind, placebo-controlled trial	73 children, 7–13 years old, 63 children completed the study (*n* = 39 EFA supplement versus 39 placebo vitamin C)	Both treatments ameliorated some ADHD symptoms. No difference in efficacy between treatments	Improvement in behavioral, sensory, and cognitive functions	No adverse events/side effects reported

Voigt et al. [42]	*Essential fatty acids* 345 mg of DHA per day (*n* = 32) or a placebo capsule (*n* = 31) for 4 months	Randomized, double-blind, placebo controlled study	54 children 6–12 years old (*n* = 27 docosahexaenoic acid (DHA) versus *n* = 27 placebo)	DHA supplementation did not significantly improve in any objective or subjective measure of ADHD symptoms		Well tolerated and no adverse effects were reported

Hirayama et al. [43]	*Essential fatty acids* DHA group: fermented soybean milk (600 mg DHA/125 mL, 3/week), bread rolls (300 mg DHA/45 g, 2/week) and steamed bread (600 mg DHA/60 g, 2/week) or placebo foods containing olive oil instead of DHA-rich fish oil for 2 weeks	Randomized, double-blind, placebo-controlled study	40 children with ADHD 6–12 years old (*n* = 20 docosahexaenoic acid (DHA) versus *n* = 20 placebo)	DHA supplementation did not improve ADHD-related symptoms		No serious side effects were reported in the study

Konofal et al. [44]	*Iron* 80 mg ferrous sulfate tablets or placebo once daily in the morning for 12 weeks	Randomized, double-blind, placebo-controlled, pilot trial	23 ADHD children with low serum ferritin level (<30 ng/mL) 5–8 years old (*n* = 18 iron versus *n* = 5 placebo)	Improvement of hyperactive/impulsive and inattentive symptoms in the ADHD rating scale	Iron is a cofactor in the synthesis of both norepinephrine and dopamine	Minor side effects were reported, such as nausea, constipation, and abdominal pain

Mousain-Bosc et al. [45]	*Vitamin B6 and magnesium* ADHD children: magnesium-vitamin B6 (Mg-B6) regimen (6 mg/kg/d Mg, 0.6 mg/kg/d vit-B6) for six months Controls did not receive Mg-B6	Open study	76 children (mean age: 6.9 years; 13 girls and 27 boys) (40 ADHD children & 36 healthy children)	Attenuation of hyperactivity and aggressiveness School attention was also improved	Vitamin B6 facilitates the production of the serotonin Magnesium is a nonspecific inhibitor of calcium and NMDA channels Magnesium can influence catecholamine signaling	No reported side effects

Bilici et al. [46]	*Zinc* 150 mg zinc sulfate or 150 mg sucrose (placebo) daily for 12 weeks	Randomized, double-blind, parallel-group placebo-controlled trial	400 children 6–14 years old (*n* = 202 zinc versus *n* = 198 placebo)	Zinc sulfate better than placebo in decreasing hyperactivity and impulsivity and improving socialization, but not inattention	Increased zinc levels necessary for cognitive development	No serious side effects reported. Metallic taste was a common complaint

Akhondzadeh et al. [47]	*Zinc* Zinc sulfate (55 mg/day) + methylphenidate (1 mg/kg/day) or sucrose (placebo) 55 mg + methylphenidate (1 mg/kg/day) for 6 weeks	Randomized, double-blind, clinical trial	44 children, 5–11 years old (*n* = 22 methylphenidate + zinc versus *n* = 22 methylphenidate + placebo)	Significantly greater treatment effects (as per parent and teacher rating scale scores) in zinc sulfate with methylphenidate treatment over placebo with methylphenidate	Zinc regulates dopamine function indirectly, through its action on melatonin	Nausea and metallic taste were common complaints. Overall, it was well tolerated

Arnold et al. [48]	*Zinc* Zinc_1: 15 mg/day (once a day) or Zinc_2: 30 mg/day (twice a day) or placebo (8 weeks); amphetamine 5–15 mg/daily (based on the weight) Duration of experiment was 13 weeks (8 weeks controlled + 5 weeks amphetamine add-on)	Randomized, double-blind, placebo-controlled, pilot trial	52 children 6–14 years old (*n* = 20 Zinc_1 or *n* = 8 Zinc_2 versus *n* = 24 placebo)	No appreciable difference between both dosages of zinc and placebo		Gastrointestinal discomfort reported by 1 patient

**Table 3:** Clinical trials demonstrating efficacy of combination therapy of botanical agents and herbs/supplements with methylphenidate in treating ADHD.

Study		Methods	Participants	Outcomes	Comments
Lyon et al. [49]	*Ginkgo biloba and Ginseng* *Ginkgo biloba* extract (50 mg) plus American ginseng, *Panax quinquefolium* (200 mg) twice/day (combination product)	Open, pilot study	36 children, 3–17 years old with ADHD	Improvement of ADHD symptoms (hyperactivity, impulsiveness, and anxiety)	Five participants reported adverse events (increased ADHD symptoms, aggressiveness, sweating, headache, and tiredness), only 2 considered related to the study

Wang et al. [50]	*Jingling oral and methylphenidate* Methylphenidate (10–40 mg/d)	Randomized, blinded study	*n* = 50 ADHD children with transient tic disorder	Significant improvement of ADHD symptoms, as well as tics	Combination therapy more effective than methylphenidate alone in improving ADHD and tic symptoms

Ding et al. [51]	*Yizhi and methylphenidate*	Randomized, methylphenidate-controlled trial	210 children with hyperkinetic syndrome	Significant improvement in ADHD symptoms in those taking combination therapy compared to either given as monotherapy	Yizhi had fewer side effects when given alone or in combination than methylphenidate

Akhondzadeh et al. [47]	*Zinc sulfate and methylphenidate*	Randomized, double-blind, and methylphenidate + placebo controlled trial	44 children (26 boys, 18 girls), 5–11 years old with ADHD	Improved parent and teacher rating scale scores for those supplemented with zinc sulfate as an adjunct	Side effects reported: anxiety, loss of appetite, nausea, headache, abdominal pain, insomnia, and metallic taste
